# Thioredoxin Network in Plant Mitochondria: Cysteine S-Posttranslational Modifications and Stress Conditions

**DOI:** 10.3389/fpls.2020.571288

**Published:** 2020-09-23

**Authors:** María Carmen Martí, Ana Jiménez, Francisca Sevilla

**Affiliations:** Abiotic Stress, Production and Quality Laboratory, Department of Stress Biology and Plant Pathology, Centre of Edaphology and Applied Biology of Segura, Spanish National Research Council, Murcia, Spain

**Keywords:** abiotic stress, mitochondria, persulfidation, redox regulation, S-glutathionylation, S-nitrosation, S-oxidation, thioredoxin

## Abstract

Plants are sessile organisms presenting different adaptation mechanisms that allow their survival under adverse situations. Among them, reactive oxygen and nitrogen species (ROS, RNS) and H_2_S are emerging as components not only of cell development and differentiation but of signaling pathways involved in the response to both biotic and abiotic attacks. The study of the posttranslational modifications (PTMs) of proteins produced by those signaling molecules is revealing a modulation on specific targets that are involved in many metabolic pathways in the different cell compartments. These modifications are able to translate the imbalance of the redox state caused by exposure to the stress situation in a cascade of responses that finally allow the plant to cope with the adverse condition. In this review we give a generalized vision of the production of ROS, RNS, and H_2_S in plant mitochondria. We focus on how the principal mitochondrial processes mainly the electron transport chain, the tricarboxylic acid cycle and photorespiration are affected by PTMs on cysteine residues that are produced by the previously mentioned signaling molecules in the respiratory organelle. These PTMs include S-oxidation, S-glutathionylation, S-nitrosation, and persulfidation under normal and stress conditions. We pay special attention to the mitochondrial Thioredoxin/Peroxiredoxin system in terms of its oxidation-reduction posttranslational targets and its response to environmental stress.

## Introduction

Reactive oxygen species (ROS) are considered unavoidable byproducts of aerobic metabolism. Mitochondria play a pivotal role in plant cells providing the energy in the form of adenosine triphosphate (ATP) during oxidative phosphorylation, but also providing intermediates of the tricarboxylic acid cycle (TCA) for major biosynthetic pathways. Mitochondria are also one of the main cellular ROS source but also a source and a target of nitric oxide (NO) and derived reactive nitrogen species (RNS) (Foyer and Noctor, 2013; Lázaro et al., 2013; Albert et al., 2017; Jayawardhane et al., 2020). Balanced ROS and RNS production and scavenging is an essential characteristic of mitochondrial function for ensuring cellular growth and plant maintenance. Local changes of ROS and RNS homeostasis mediate downstream signaling events *via* interactions with individual proteins and signaling. Similar to ROS and RNS, a new player in mitochondrial signaling is H_2_S (Filipovic and Jovanović, 2017). Proteins sense redox changes and transmit information by posttranslational modifications (PTMs) of cysteine sensitive thiols, which are oxidized to proteins disulfide, trisulfides, sulfenic, sulfinic or sulfonic acid derivates, as well as S-glutathionylated and S-nitrosated forms. Additionally, H_2_S does not spontaneously react with reduced thiol groups but it can react with oxidized ones leading to protein persulﬁdation (Sevilla et al., 2015a; Wang et al., 2018; Huang et al., 2019).

In the process of ROS elimination and redox signal integration, mitochondria contain antioxidants enzymes such as Mn-superoxide dismutase (Mn-SOD) and ascorbate dependent peroxidase (APX) (Jiménez et al., 1997; Chew et al., 2003) as well as redox sensitive proteins, which are classified into two classes: redox sensors, including peroxiredoxin (PRX) and glutathione peroxidase-like (GPXL) and redox transmitters, containing thioredoxin (TRX) and glutaredoxin (GRX). However, in contrast to that occurring in plastids, cytosol, and nucleus, the TRX/PRX and glutathione/GRX systems are not very well studied in mitochondria. The scarce information about TRX/PRX system in mitochondria is striking, taking into account that posttranscriptional/translational regulation is a likely determinant of the mitochondrial stress response as well as the role of this redox system as sensor and initiator of signaling cascades in a variety of processes and stress conditions (Sevilla et al., 2015a; Zannini et al., 2018).

This review is focused on summarizing the information about ROS, RNS and H_2_S dependent mitochondrial PTMs, as regulatory mechanisms of plant mitochondria function under normal and stress conditions. We focus on the main enzymes from the major biological processes occurring in the mitochondria such us the ETC, the TCA and photorespiration and on how PTMs affecting cysteine residues (S-oxidation, S-glutathionylation, S-nitrosation, and persulfidation) exert their regulatory mechanisms on plant mitochondrial enzymes. We pay special attention to the mitochondrial TRX/PRX system in terms of its posttranslational control and its response to environmental stress.

## Mitochondrial ROS, RNS, and H_2_S Generation

### ROS Generation in Mitochondria

Oxygen is essential for the aerobic life and due to its chemical feature as a molecule containing two unpaired electrons it can suffer monovalent reduction generating ROS with a bimodal action. Their beneficial aspects are related to the effect on the cellular redox state and the role in signaling cascades while their detrimental aspect is related to an overproduction or insufficient endogenous antioxidant defenses which can damage all kind of cellular structures leading, in the worst case scenario, to cell death (Mhamdi and Van Breusegem, 2018). As signaling molecules, ROS have a special importance during developmental and physiological processes in plants. A redox compartmentalization exists for a proper function of ROS-dependent signaling pathways and ROS production, which depend on the reaction with other reactive species and interaction with antioxidants and scavengers (Waszczak et al., 2018; Foyer et al., 2020). Mitochondria generate energy in form of ATP by oxidative phosphorylation during glucose metabolism, a process coupled with mitochondrial respiration through a generated transmembrane potential *via* mitochondrial complexes I to IV of the electron transport chain (ETC), with the final result of the four-electron reduction of O_2_ to H_2_O. ROS are generated as byproducts after electron leaking at complex I and III (Hernández et al., 1993; Noctor et al., 2007) and under specific conditions at complex II during the reverse electron transport (Turrens, 2003). Thus, ROS such as superoxide anion (O_2_^·−^), hydrogen peroxide (H_2_O_2_) and hydroxyl radical (HO.) are generated after one, two and three-electron reduction, respectively. A role for complex II in H_2_O_2_ production has been reported to be even more important that complex I with a special influence under stress conditions, and also ubiquinone pool might serve as ROS site in plant mitochondria (Umbach et al., 2005; Gleason et al., 2011). A high proportion of NADH/NAD^+^ and/or ADP/ATP can increase the electron transport contributing to a high membrane potential, which is correlated to a higher reduction of the ETC components, increasing the probability of electron leakage to O_2_ and thus increasing ROS production. In the presence of transition metal ions, the more reactive HO^·^ is formed. The O_2_^·−^ generated in the mitochondrial matrix is rapidly dismutated to H_2_O_2_ by Mn-SOD contributing to the ROS production in the organelle. This H_2_O_2_ is scavenged by APX (Jiménez et al., 1997; Jiménez et al., 1998) and PRXIIF (Barranco-Medina et al., 2007). The plant ETC contains other enzymes which do not contribute to the proton gradient and act as security oxidation valves limiting ROS production: type II NAD(P)H-dehydrogenases can avoid the electron transport from complex I and II to ubiquinone while alternative oxidase (AOX) couples the ubiquinol oxidation to the O_2_ reduction to water and dissipation of energy in form of heat (Rasmusson and Wallström, 2010; Del-Saz et al., 2017). The increased and/or decreased expression of AOX along with the inhibition of its activity, have been considered as important factors in the establishment of the mitochondrial generation of ROS in signal transduction pathways (Vishwakarma et al., 2015) with the induction of AOX described as a mitochondrial retrograde signaling model (Maxwell et al., 1999; Lázaro et al., 2013). Evidences obtained with respiratory inhibitors and/or mutants have shown that fully functional mitochondria are also determinant for optimal photosynthesis. That dependency was linked to both cytochrome c oxidase (COX) and AOX pathway as well as to uncoupled protein (UCP) activity, which may modify the oxidation rate of ETC substrates by an increase of ubiquinol oxidation when the electrochemical proton gradient decreases across the inner membrane (Millar et al., 2011). Together with AOX, the terminal enzyme of ascorbate (ASC) biosynthesis, L-galactone-1,4-lactone dehydrogenase (GLDH), has been described to be a dual-function enzyme as structural assembly factor of complex I and as associated to the cytochrome respiratory pathway (Schimmeyer et al., 2016; Rodríguez-Ruiz et al., 2017) allowing the synthesis of ASC when coupled as an alternative electron donor for the respiratory chain. In this way, this protein may have an specific role under conditions of possible inhibition of components of the TCA as occurring under oxidative stress (Dumont and Rivoal, 2019). Interestingly, the last step of ASC biosynthesis does not produce H_2_O_2_ as it happens in animals where the activity is carried out by an oxidase instead of a dehydrogenase, altering the cellular redox state (reviewed by Fenech et al., 2019).

### RNS Generation in Mitochondria

Nitric oxide (NO) refers to its nitrosyl radical (^·^NO). In many species, including plants, NO is a key signalling molecule that plays a role in a large number of biological processes (Kolbert et al., 2019) due to its chemistry. Reactive nitrogen species (RNS) are a family of molecules derived from NO, such as S-nitrosothiols (SNOs), S-nitrosoglutathione (GSNO), peroxynitrite (ONOO-), nitrogen dioxide (NO_2_), and nitro-fatty acids (NO_2_-FA), among others (Romero-Puertas et al., 2008; Martínez-Ruiz et al., 2013; Mata-Pérez et al., 2017).

In mitochondria both, an oxidative and a reductive pathway have been described for the NO generation. Regarding the oxidative route involving an L-Arg-dependent NOS-like activity, a mitochondrial Arabidopsis NO synthase 1 (AtNOS1) implicated in mitochondrial biogenesis was identified (Guo and Crawford, 2005). However, this enzyme was later on characterized as a small GTPase and not a NO synthase, being renamed as AtNOA1 (nitric oxide associated 1). In contrast to animals, a NOS-like enzyme has not been identified in plants (Astier et al., 2018; Kolbert et al., 2019). The reductive route of NO generation is dependent on nitrite and occurs in the inner membrane probably *via* cytochrome c oxidase (CCO) and/or reductase, generating ATP under hypoxia (Jayawardhane et al., 2020). Using different inhibitors, the involvement of the respiratory chain mainly complex III, CCO, and AOX was later reported although with clear results for CCO again under hypoxia (Gupta and Igamberdiev, 2016). The NO generated, can accept or loose an electron to generate nitrosyl anion (NO^-^) or the nitrosonium cation (NO^+^) or other RNS including higher oxides such as NO_2_ and N_2_O_3_, or it can react immediately with superoxide originated from ETC, to form peroxynitrite (ONOO−) (Leitner et al., 2009). NO can also react with GSH to form GSNO, considered as an *in vivo* reservoir of NO.

### H_2_S Generation in Mitochondria

Sulfur is an essential macronutrient, taken up as sulfate and assimilated into cysteine. Volatile H_2_S is an inflammable gas considered as a pollutant but also as a new signaling molecule in both plants and animals (Filipovic and Jovanović, 2017). In plants it has been involved in multiple processes including response to abiotic stresses, regulation of photosynthesis, stomatal closure or autophagy (Aroca et al., 2018; Zhou et al., 2020). Opposite to ROS and RNS, H_2_S does not spontaneously react with reduced thiol groups, but it can react with oxidized ones leading to protein S-sulfhydration (now named persulﬁdation) (Paul and Snyder, 2012; Dumont and Rivoal, 2019). The increasing interest on H_2_S resides, among others, in that when administered exogenously, it has a positive effect in regulating plants adaptation (Zhang et al., 2009; Jin et al., 2017; Aroca et al., 2018), although the mechanism is not fully understood.

In plant systems, H_2_S is generated mainly in chloroplasts *via* the photosynthetic sulfate-assimilation pathway (García et al., 2015) although in mitochondria, the enzyme cyanoalanine synthase c1 (CAS-C1) also generates sulﬁde (Álvarez et al., 2012). Other enzymes described in mitochondria are certain desulfydrases which decomposes D/L-cysteine into pyruvate, H_2_S, and NH_3_ (Riemenschneider et al., 2005).

## Post-Traslational Modifications Affecting Cysteine Residues: S-Oxidation, S-Glutathionylation, S-Nitrosation, and Persulfidation

### S-Oxidation

**Figure 1:**
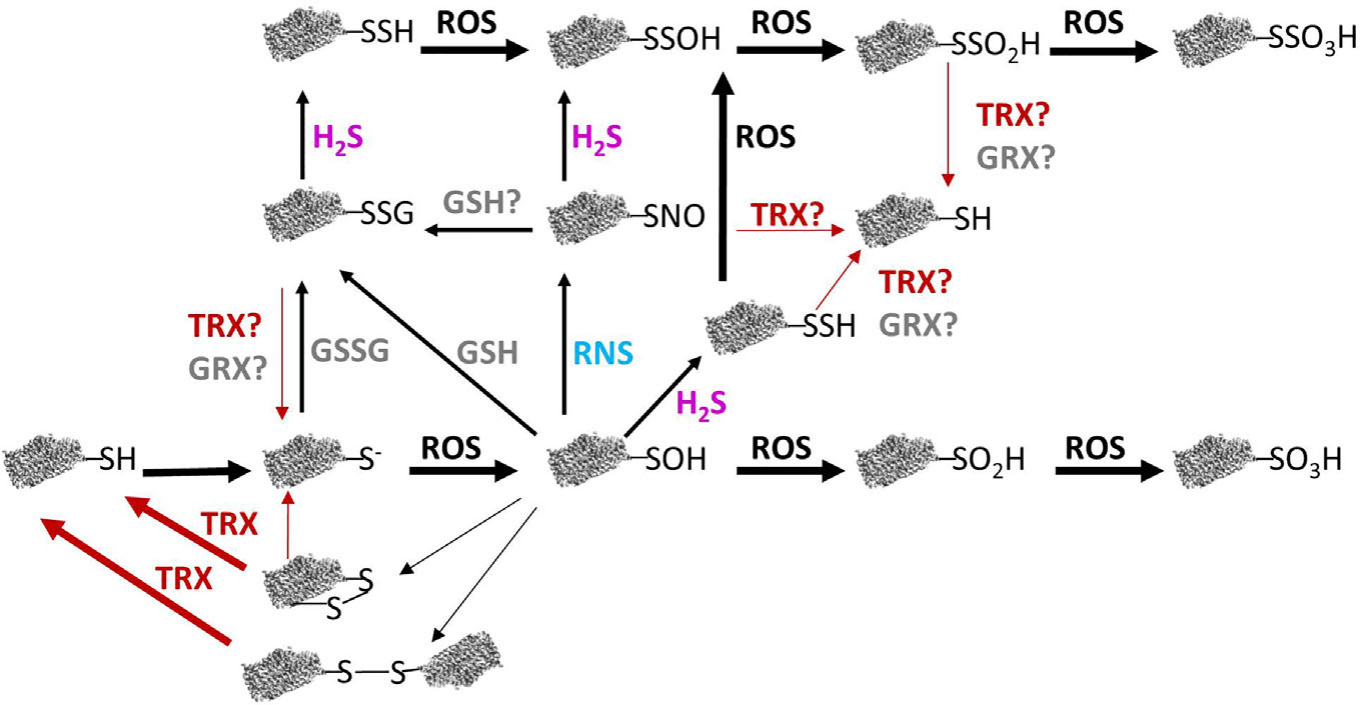
Relation between redox modiﬁcations of protein cysteine thiols and their reversibility in plant mitochondria. Reactive oxygen species (ROS)-mediated oxidation of protein thiols leads to different redox modifications. Reactive nitrogen species (RNS) can produce S-nitrosation of cysteine residues and GSH and GSSG provoke S-glutathionylation whereas H_2_S can lead to persulfidation. Some of these modifications may be reversed by TRX or GRX (see text for details). GRX, glutaredoxin; GSH, reduced glutathione; GSSG, oxidized glutathione; H_2_S, hydrogen sulﬁde; RNS, reactive nitrogen species; ROS, reactive oxygen species; R-S−, thiolate anion; R-SH, reduced thiol; R-SNO, S-nitrosation; R-SOH, sulfenic acid; RO_2_H, sulfinic acid; RO_3_H, sulfonic acid; R-SSG, S-glutathionylation; R-SSH, persulfidation; R-SSOH, persulfenic acid; R-SSO_2_H, persulfonic acid; R-SSO_3_H, persulfinic acid; R-SS-R, disulﬁde bond; TRX, thioredoxin.

The –SH group of cysteine has a pKa of about 8.3 in free amino acids. However, in oxidoreductases proteins, such as TRXs and glutaredoxins (GRXs) this pKa is lower, because it is affected by the surrounding micro-environment (Mailloux et al., 2014). Therefore, the –SH group of this type of cysteine residues will appear predominantly as more reactive thiolate and will react with close -SH groups to form a disulfide bridge (Meyer et al., 2012; Sevilla et al., 2015a). Additionally, –SH cysteine residues will suffer different oxidation states in response to redox signals mediates by ROS (**Figure 1**).

The key player ROS that provokes oxidative reactions is H_2_O_2_, due to three main characteristics: reactivity with cysteine, prolonged half-life relative to other ROS, and capacity to diffuse through membranes. The significance of the oxidation produced by H_2_O_2_ on cysteine residues depends on the levels of H_2_O_2_. At high levels of H_2_O_2_, proteins can be oxidized irreversibly and lost their ability to function. Thus, H_2_O_2_ levels are tightly controlled by antioxidant systems to avoid overoxidation of functional proteins. In this sense, enzymes as catalase, multiple Cys-based peroxidases including PRXs, GPX, and those enzymes of ASC-GSH cycle, exert a fine control of H_2_O_2_ cellular levels (Dietz, 2011; Foyer and Noctor, 2011; Lázaro et al., 2013).

As the levels of H_2_O_2_ rise, it oxidizes the thiolate anion of protein cysteine residues to the sulfenic form (Cys–SOH). The sulfenic form can form an intra-or intermolecular disulfide bond by reaction with a neighboring thiolate or can be reduced and reverted to the thiolate form. A typical example of this kind of protein is the PRX, which by the action of TRXs and/or GRXs can be converted back to the reduced thiol group (Dietz, 2011; Sevilla et al., 2015a). Cysteine residues involved in dithiol-disulfide redox exchange frequently are located at catalytic sites of enzymes or are cysteines involved in metal binding and usually show a high degree of conservation. This dithiol-disulfide modification is transient and can act as a molecular switch altering protein activity, conformation, subcellular localization, and/or binding properties (Van Ruyskensvelde et al., 2018). In fact, protein cysteine sulfenylation is considered to play a key role in sensing and redox signalling (Huang et al., 2019). Alternatively, Cys–SOH can react with low-molecular weight thiols, such as GSH, to form S-glutathionylated disulfides. If the levels of H_2_O_2_ increases, the sulfenic form can suffer two more oxidation steps and become sulfinic (SO_2_H) or sulfonic (SO_3_H) species. The sulfinic form can be reduced by sulfiredoxin (SRX) through an ATP-dependent reaction (Iglesias-Baena et al., 2011), however the hyperoxidized sulfonic form, to date, has been shown to be irreversible (**Figure 1**).

### S-Glutathionylation

The presence of GSH in plant mitochondria (Jiménez et al., 1997; Fernández-García et al., 2009; Foyer and Noctor, 2011) allows the posttranslational modification (PTM) of proteins *via* reversible S-glutathionylation of protein cysteine residues. S-glutathionylation can occur by spontaneous disulfide bond formation between the cysteine sulfenic form and GSH or a thiolate form derived from a reduced cysteine with GSSG, being the extend of the reactions dependent on the GSH/GSSG ratio in mitochondria, which is usually high in normal conditions (Dumont and Rivoal, 2019) (**Figure 1**). S-glutathionylation can be considered as a defense mechanism against the overoxidation of cysteine residues during oxidative stress that decreases the GSH/GSSG ratio (Zechmann, 2014; Sevilla et al., 2015b). After glutathionylation, disulfide bonds may be formed with another protein thiol and both forms can be reversed by GSH, GRX, or TRX as it has been described in animal systems (Beer et al., 2004) (**Figure 1**). In plants, several TRXs *h* present a de-glutathionylation activity although with a lower efficiency than GRXs as demonstrated for Arabidopsis cytosolic glyceraldehyde-3-phosphate dehydrogenase (Bedhomme et al., 2012), explaining the absence of GRX in plant mitochondria with de-glutathionylation activity (Zannini et al., 2018). In fact, mitochondrial TRX *h* undergoes glutationylation and the thiolation alters its redox potential as identified by MS (Gelhaye et al., 2004).

### S-Nitrosation

The presence of RNS in mitochondria and the PTMs that they produce, allow mitochondria to be involved in many biological processes. RNS exert their role mainly by protein tyrosine nitration, metal nitrosylation and S-nitrosation (Romero-Puertas et al., 2008; Camejo et al., 2013; Corpas et al., 2013; Camejo et al., 2015; Lamotte et al., 2015). Tyrosine nitration is mediated by ONOO- and NO_2_ which are generated respectively, by reaction of O_2_^.-^ and O_2_ with NO. During this process, a 3-nitrotyrosine group is produced by addition of a nitro group at the ortho position of the phenolic group of tyrosine (Ischiropoulos et al., 1992). During metal nitrosylation, NO binds to transition metals in metalloproteins (Keilin and Hartree, 1937). Finally, the most important pathway by which RNS execute their action is by protein S-nitrosylation. S-nitrosylation can occur by different mechanisms depending of the NO source, all of them resulting in the addition of a NO group to a cysteine residue in the target protein (**Figure 1**). During S-nitrosation, the S-nitrosothiol can be formed through NO reaction with thiyl radicals formed from one electron oxidation of thiolates *via* NO_2_ or NO_2_ and NO can react to form N_2_O_3_, which reacts with the cysteine thiolate (Hess et al., 2005). Additionally, S-nitrosation can occur *via* trans-nitrosylation, by which an S-nitrosylated group transfers its NO to another thiolate group or to GSH to form the NO reservoir GSNO, which in turn can also transfer its NO to S-nitrosylate proteins or its GSH to S-glutationylate proteins. In fact, GSNO is a mobile NO pool which effectively transduces NO signalling. To do that, GNSO levels are tightly controlled by its production and degradation. Previously we mentioned that GSNO is formed by the transfer of NO to GSH. GSNO degradation can be *via* non-enzymatic and *via* GSNO reductase (GSNOR) enzymatic activity (Lindermayr, 2018). S-nitrosylation and therefore, GSNOR, can regulate a large amount of cellular functions and signaling events due to the capacity of alter the activity, stability, conformation, interactions with other molecules or subcellular localization of the S-nitrosated proteins, playing an essential role to protect cells under nitrosative stress (Sevilla et al., 2015a; Romero-Puertas and Sandalio, 2016; Corpas et al., 2019a).

### Persulfidation

H_2_S can exert its function through persulfidation, a PTM affecting the thiol group of cysteine (-SH) in proteins to be modified into a persulﬁde group (-SSH) (Gotor et al., 2019; Sandalio et al., 2019). H_2_S reacts with either disulfides or sulfenic acids to yield persulfides which are highly reactive to ROS, being oxidized to perthiosulfenic acids (–SSOH), perthiosulfinic (–SSO_2_H), and perthiosulfonic (–SSO_3_H) acids (**Figure 1**). Oxidized persulfides, can be then reduced back to their thiol forms by TRXs or GRXs (Wedmann et al., 2016; Zhou et al., 2020), so the presence in plant mitochondria of TRX*o*1 (Martí et al., 2009) may be a key component of the action of this PTM in the organelle, allowing the recycling and reusing of H_2_S by the cell.

## Mitochondrial Targets of S-Oxidation, S-Glutathionylation, S-Nitrosylation, and Persufidation

**Figure 2:**
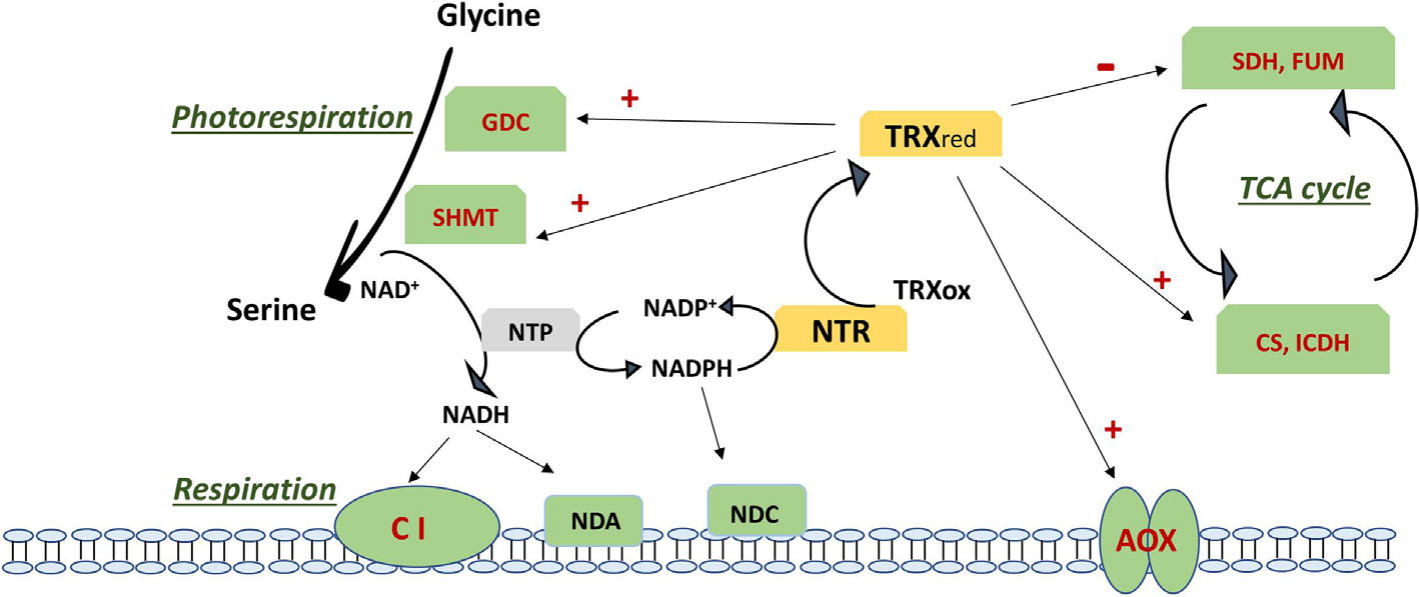
Regulation of photorespiration, respiration and TCA cycle *via* the action of thioredoxin on specific targets (in red). Negative regulation is shown as – and positive regulation as +. TCA cycle is regulated by inhibition of SDH and FUM and the activation of CS and ICDH. Respiration is regulated *via* activation of AOX and by the NADH generated by an active photorespiration with the Gly to Ser conversion regulated by the TRX-mediated activation of GDC an SHMT, which generates an increase in NADH. NADH excess can be oxidised *via* complex I and internal dehydrogenase NDA or can be transhydrogenated by NTP with formation of NADPH. NADPH is used by NTR to reduce back TRX from its oxidised form or can be oxidized *via* the internal dehydrogenase NDC. Updated scheme based on the previously reported one (Bykova and Igamberdiev, 2016). AOX, alternative oxidase; CI, complex I; CS, citrate synthase; FUM, fumarase; GDC, glycine decarboxylase complex; ICDH, isocitrate dehydrogenase; NDA, internal NADH dehydrogenase; NDC, internal NADPH dehydrogenase; NTP, NAD(P)transhydrogenase; NTR, NADPH thioredoxin reductase; Ox, oxidized; Red, reduced; SDH, succinate dehydrogenase; SHMT, serine hydroxymethyl transferase; TCA, tricarboxylic acid; TRX, thioredoxin.

**Figure 3:**
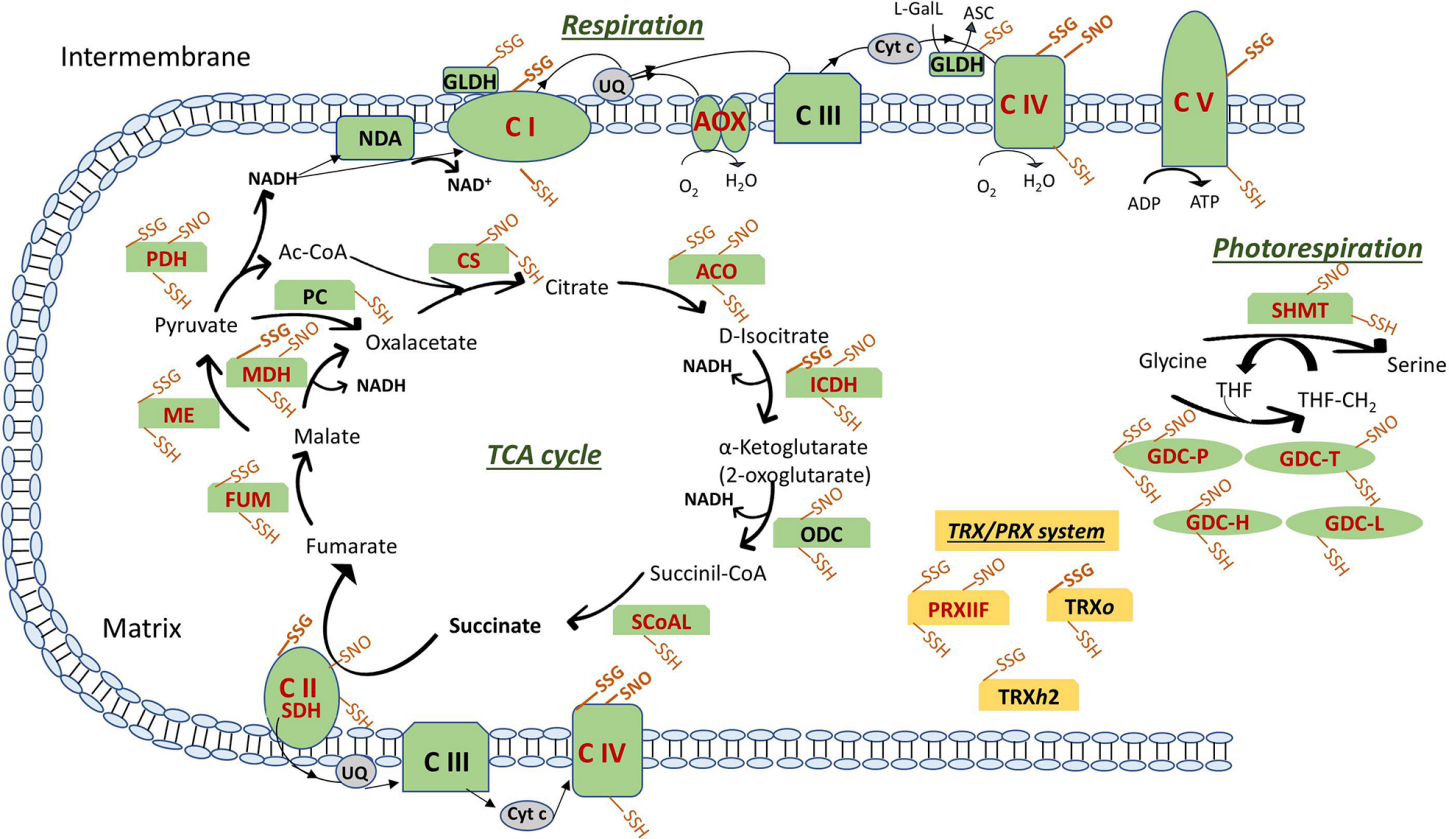
Posttranslational modiﬁcations of cysteine residues of ETC components, TCA cycle, photorespiration enzymes and TRX/PRX system in plant mitochondria. Modifications are shown as SSG (S-glutathionylation), SNO (S-nitrosylation) and SSH (Persulfidation). In bold the modifications described only in mitochondria from animal systems. Enzymes are in red when reported as thioredoxin targets (see text for details and references). ASC, ascorbate; ETC, electron transport chain: NDA, internal NADH dehydrogenase; Complex (C) I: NADH Dehydrogenase; CII: SDH, succinate dehydrogenase; CIII: Cytochrome (Cyt) b-c reductase; CIV: Cyt c oxidase; CV: ATP synthase; AOX: alternative oxidase; GLDH, L-galactono-1,4-lactone dehydrogenase; L-GalL, L-galactono-1,4-lactone; UQ, ubiquinone. TCA, tricarboxylic acid cycle: ACO, aconitase; CS, citrate synthase; FUM, fumarase; ICDH, isocitrate dehydrogenase; MDH, malate dehydrogenase; ME, malic enzyme; ODC, 2-oxoglutarate decarboxylase; PC, pyruvate carboxylase; PDH, pyruvate dehydrogenase; SCoAL, Succinyl-CoA synthetase. Photorespiration: GDC, glycine decarboxylase (P, T, H and L proteins); SHMT, serine hydroxymethyltransferase; THF, tetrahydrofolate; THF-CH2, N-5, N-10-methylenetetrahydrofolate. TRX/PRX system: PRX, peroxiredoxin; TRX, thioredoxin.

In this review, we focus on the cysteine S-oxidation described for mitochondrial TRX targets and other cysteine PTMs that affect those proteins and/or proteins that are key players in the TCA, the ETC and photorespiration (**Figures 2**, **3**).

### Targets of S-Oxidation

TRXs are small ubiquitous proteins with a conserved CPGC motif in the active center enabling the reduction of disulfides bonds of specific target proteins through a dithiol-disulfide exchange mechanism. In Arabidopsis, at least 20 *TRX* genes have been reported, classified into eight subgroups (*f, h, m, o, s, x, y*, and z-type) (Alkhalfioui et al., 2008; Meyer et al., 2012). The mitochondrial TRX isoforms belong to the TRX*h* and TRX*o* groups. The presence of TRX and NADPH-TRX reductase (NTR) activities was initially detected in plant mitochondria (Marcus et al., 1991; Konrad et al., 1996), but the native proteins were not identified and characterized. Laloi et al. (2001) identified *AtTRX-o1* and *AtTRX-o2* genes encoding two TRX*o* isoforms in Arabidopsis, being TRX*o*1 unequivocally present in mitochondria whereas the TRX*o*2 cellular location is still not clear (Zannini et al., 2018). Later on, Gelhaye et al. (2004) found that poplar mitochondria also contain a TRX isoform belonging to the TRX*h* type (PtTRX*h*2), earlier identified in the cytosol. More recently, a new *TRXo1* gene was identified in *Pisum sativum* L. and located in both mitochondria and nucleus under physiological non –stress conditions (Martí et al., 2009). TRXs regeneration relies on the NTRA and/or NTRB being both genes expressed in mitochondria and cytosol, although *NTRA* has been also found in the nucleus (Reichheld et al., 2005).

In Arabidopsis, a recent structure-function analysis of both TRX*o* isoforms, has demonstrated that both recombinant proteins expressed in *E. coli* bind a Fe-S cluster. In TRX*o*2, the Fe-S cluster ligation depends on the cysteine residues found in the active conserved motif, but the physiological relevance of this observation remains unclear. Furthermore, it was observed a possible connection between TRXs *o* with the mitochondrial Fe-S cluster as both TRXs *o* shown the same capacity to reduce oxidized nitrogen-fixation-subunit-U 4 (NFU4) or NFU5. These NFU proteins were previously isolated as putative TRX partners and known to participate in the maturation of certain mitochondrial Fe-S proteins (Braymer and Lill, 2017; Ciofi-Baffoni et al., 2018).

An important advance in the research on the physiological role of TRX in plants has been carried out in the last two decades, but the specific role in mitochondria remains to be fully elucidated and only Arabidopsis and pea TRX*o*1 have been deeper studied. Several redox-proteomics studies using TRX affinity chromatography approaches have identified potentially redox regulated proteins in plant mitochondria. Thus, the TRX*o*1 function has been related with mitochondrial processes including, the respiratory AOX pathway, the detoxification of ROS *via* mitochondrial PRXIIF, the TCA cycle enzymes, the energy (ATP) synthesis and protein translation (Balmer et al., 2004; Barranco-Medina et al., 2008; Martí et al., 2009; Yoshida et al., 2013). In parallel, transcriptional regulation and redox regulation *via* TRXs is believed to play a crucial role in adapting photorespiration to changing environmental conditions (Reinholdt et al., 2019a).

Over-reducing conditions of mitochondrial ETC activate AOX by promoting the conversion of its dimeric oxidase form into the active reduced dimers as a consequence of the reduction of a functional disulfide bond (Day et al., 1994). Although AOX protein has not been identified as a putative mitochondrial TRX target using affinity chromatography, it has been shown to be activated by TRX-dependent reversible thiol-disulfide switch, being the mitochondrial TRX the candidate to this redox regulation (Lázaro et al., 2013; Selinski et al., 2018). AOX reduction and activation by mitochondrial PtTRX*h*2 and its effector pyruvate was initially described (Gelhaye et al., 2004; Umbach et al., 2005). Further, the addition of recombinant NADPH/NTR/PsTRX*o*1 to isolated pea and soybean mitochondria preparations induced both the reduction of AOX homodimers through the thiol redox switch of the protein and the activation of the AOX capacity (Martí et al., 2009). A similar induced AOX thiol redox switch has been linked to recombinant TRX*o*1 system in Arabidopsis and recently in thermogenic skunk cabbage (Yoshida et al., 2013; Umekawa and Ito, 2019). However, aside from these studies in isolated mitochondria, there is still no conclusive evidence showing that AOX is indeed regulated by the TRX system *in vivo* (Sevilla et al., 2015a; Geigenberger et al., 2017) (**Figure 2**).

PRXIIF is considered as partner of TRX*o* and was captured by PsTRX*o*1 affinity chromatography using mitochondrial preparations (Martí et al., 2009). PRXs are dimeric thiol-based peroxidases involved in H_2_O_2_ and alkyl hydroperoxide reduction to water and the corresponding alcohol, respectively. PRXs play an important role as redox sensors, antioxidant defense and in signaling. The mitochondrial location of Arabidopsis and pea PRXIIF was reported by Finkemeier et al. (2005) and Barranco-Medina et al. (2007), respectively. A biochemical characterization analysis, the use of recombinant proteins and microcalorimetric (ITC) titrations techniques reported the binding of PsTRX*o*1 and PsPrxIIF, sustaining a role of TRX*o*1 as a physiological electron donor to PrxIIF (Barranco-Medina et al., 2007; Barranco-Medina et al., 2008). A common characteristic for PRXs from plants and mammalians is the presence of at least one conserved cysteine at the active site called peroxidatic cysteine Cp (Barranco-Medina et al., 2007; Dietz, 2011; Toledo et al., 2011). Under the normal catalytic mechanism, reaction of Cp with H_2_O_2_ is very fast generating the sulfenic acid (Cys-SOH), which then form an inter-or intramolecular disulfide bond with a resolving cysteine (Cr) (Toledo et al., 2011). The disulfide or sulfenic from of PRXs is subsequently reduced by TRX/GRX (Barranco-Medina et al., 2009; Meyer et al., 2012). The cysteine sulfenic form may be overoxidized to the sulfinic form (Cys-SO_2_H), which can be reduced by SRX in the presence of ATP. Finally, a thiosulfinate intermediate between both PRX and SRX is formed (Iglesias-Baena et al., 2010). The regeneration step is catalyzed by a thiol reductant like NTR/TRX and/or GSH (Iglesias-Baena et al., 2011; Liebthal et al., 2018). In pea and Arabidopsis plants, the small thiol reductase protein, SRX has also been described to be localized in mitochondria and in chloroplasts (Iglesias-Baena et al., 2010; Iglesias-Baena et al., 2011). In mitochondria, SRX presents a broader specificity than in chloroplasts, catalyzing the retro-reduction of hyperoxidized (sulfinic) atypical mitochondrial PRXIIF and atypical human PRXV (Rey et al., 2007; Iglesias-Baena et al., 2010; Iglesias-Baena et al., 2011).

Some TCA enzymes were described to be highly susceptible to oxidative stress as citrate synthase (CS) and isocitrate dehydrogenase (ICDH) (Geigenberger et al., 2017). CS activity in Arabidopsis was demonstrated to be inhibited by oxidation (Stevens et al., 1997). Later on, the mitochondrial CS isoform was also shown to be regulated by TRX*o* (Schmidtmann et al., 2014). In contrast to other TCA cycle enzymes, CS is exclusively localized in mitochondria in green tissues, so the TCA cycle cannot be bypassed *via* cytosolic isoforms in those tissues. By site-directed mutagenesis of its six cysteine residues the authors reported that oxidation inhibited enzyme activity by the formation of diverse disulfide bridges, as the partially oxidized enzyme forms large redox-dependent aggregates. TRX can cleave those intra- and intermolecular disulfide bridges, reversing the enzyme to the active state and strongly enhancing the activity (**Figure 2**). Daloso et al. (2015) described an activation by TRX*o*1 *in vitro* although an increase in the leaf enzyme activities in *trxo1* and *ntra ntrb* mutants suggested that this activation may not occur *in vivo*. Mitochondrial NAD^+^-dependent isocitrate dehydrogenase (mICDH) was also captured using TRX affinity chromatography. This enzyme forms only intramolecular disulfide bridges under oxidizing conditions (Yoshida and Hisabori, 2014). Recombinant Arabidopsis mICDH has a heterodimeric structure composed of two subunits: ICDH-c and ICDH-r and unlike yeast ICDH, it did not show adenylated-dependent enzyme activity. Upon oxidation of the subunit ICDH-r, ICDH activity was largely diminished *via* intermolecular disulfide–mediated oligomer formation of ICDH-r. A recent study has confirmed that AtTRX*o* was effective in the reduction of oxidized ICDH-r likely leading to a recovery of ICDH activity (Yoshida and Hisabori, 2014) (**Figure 2**). In the first studies using the double T-DNA mutant of Arabidopsis with downregulated *NTRA* and *B* expression (*ntra ntrb*) and the mitochondrial located thioredoxin *o*1 (*trxo1*) mutant, Daloso et al. (2015) demonstrated that the mitochondrial TRX system regulates *in vivo* the activity of different enzymes of the TCA cycle. In particular, the mitochondrial TRX*o*1 deactivates fumarase (FUM) and succinate dehydrogenase (SDH) both previously reported as potential TRX target proteins (Yoshida et al., 2013). These enzymes bear a considerable proportion of the flux control of TCA pathway, with SDH linking the TCA cycle and ETC. Curiously, TRX*o* activates the cytosolic citrate lyase. On the basis on these findings, the authors point out a role of mitochondrial TRX*o* as crucial mediator of mitochondria-cytosol cross-talk controlling carbon fluxes in the cell (Daloso et al., 2015). The enzyme ATP-citrate lyase is involved in fatty acid biosynthesis, a noncyclic mode of TCA function, using citrate exported from the mitochondria. It has been suggested that this TRX*o*1/citrate lyase regulation might be favored under reducing conditions characterized by a high NAD(P)H/NAD(P)^+^ ratio, which allows a deceleration from the TCA cycle (Møller, 2015) (**Figure 2**). Other TCA cycle enzyme that was trapped on TRX affinity columns is malate dehydrogenase (MDH). In plants, two mitochondrial NAD-dependent MDHs have been described with a role in central metabolism and redox homeostasis (Selinski et al., 2014; Huang et al., 2017). Initially, it was reported that mitMDH could be regulated *via* mitochondrial TRX*o*. However, Daloso et al. (2015) described an unaltered effect by TRX*o*1 finding similar activity in WT and *trxo1* and *ntra ntrb* mutant plants (**Figure 2**). Additionally, mitMDH activity has been shown to be regulated by adenin nucleotides (Yoshida and Hisabori, 2016).

Photorespiration or “respiration in light” links photosynthetic carbon assimilation with nitrogen and sulfur assimilation and carbon metabolism, contributing to balancing the redox state between mitochondria, chloroplasts, peroxisomes and cytoplasm. In mitochondria, a key step in photorespiration is the conversion of glycine from peroxisomes into serine which will be back to peroxisomes for its deamination (Engel et al., 2007). This step is carried out by the action of a multienzyme system composed of the complex glycine decarboxylase (GDC, with 4 subunits: P, T, L and H) and serine hydroxymethyl transferase (SHMT; Bauwe and Kolukisaoglu, 2003). The NADH generated by GDC is oxidized by the respiratory ETC leading to higher NADH/NAD^+^ ratio in the organelle and increased cytosolic ATP/ADP under photorespiratory conditions (Eisenhut et al., 2019) (**Figure 2**). GDC proteins have been identified as potential targets of mitochondrial TRX*o*1 in different plants as well as in *Synechocystis* PCC 6803. In pea, mitochondrial P and T components were identified as PsTRX*o*1 targets together with SHMT (Martí et al., 2009). More recent research has shown that, the lack of *TRXo1* caused a slow-down of GDC activity with an impaired glycine to serine turnover. Results evidenced that *TRXo1* contributes to redox-regulation of GDC due to TRX*o*-mediated redox regulation of lipoamide dehydrogenase (GDC-L or LPD), since its activity decreases in plant and cyanobacteria when reduced *via* NTRA/TRX*o*1 system. Redox regulation of LPD has also been suggested in *Chlamydomonas reinhardtii* (Pérez-Pérez et al., 2017). It was concluded that TRX*o*1 contributes to adjust photorespiration in response to environmental fluctuations *via* the regulation of GDC and possibly other mitochondrial multienzyme systems in which LPD is involved (Reinholdt et al., 2019a). Not only TRX*o*1, but also AtTRX*h*2, contributes to the redox regulation of mitochondrial photorespiratory metabolism (Da Fonseca-Pereira et al., 2019a) (**Figure 2**). AtTRX*h*2 regulates the redox state of GDC-L protein, which is altered in *trxh2* mutants *in vivo*. The recombinant TRX*h*2 can also deactivate GDC-L *in vitro*. Decreased abundance of SHMT and GDC H and L subunits as well as reduced NADH/NAD^+^ ratio, were also found.

All the described evidences allow us to conclude that a consistent overall picture is emerging indicating that central carbon metabolism is controlled by protein thiol switches that affect enzymatic activity. To determine the physiological and molecular function of mitochondrial TRX on the *in vivo* regulation of the identified targets and metabolism, plant mutants have been recently probe to be useful, therefore plants mutants needs to be analyzed further.

### Targets of Glutathionylation

Glutathionylation in cells represents the major form of S-thiolation mainly occurring under oxidative stress conditions although it may also be important under normal conditions and the exact mechanism *in vivo* is not clearly elucidated (Gao et al., 2009). Several methods have been developed to glutathionylate proteins *in vitro* and also to induce glutathionylation by oxidative stress in proteomic studies. Among them, GSSG-biotin is considered a component of oxidation because of the shift in the glutathione redox towards the oxidized state (Brennan et al., 2006). Other glutathionylating agents as GSH-biotin or biotin-amide that do not induce oxidative stress are usually used together with oxidants as diamide, ter-buthyl hydroperoxide (TBHP) or H_2_O_2_. Also, anti-GSH antibody is used to identify thiolation in protein extracts or in specific purified proteins after immunoprecipitation and western blot assay, although a major concern is related to its specificity and sensitivity (Gao et al., 2009).

Some TCA cycle enzymes have been identified as thiolated such as FUM, malic enzyme and pyruvate decarboxylase in Arabidopsis cell cultures treated with GSSG-biotin and TBHP and identification by MS (Dixon et al., 2005). Glutathionylation may be also important to modulate ACO activity under oxidative and nitrosative stress, due to the fact that apart from the cysteine thiol oxidation to sulfonic acid, using anti-GSH, the purified enzyme was shown to be glutathionylated, and both modifications decreased its activity (Han et al., 2005). Other TCA enzymes identified as glutathionylated but in different animal systems are succinate dehydrogenase (SDH) and MDH (Kil and Park, 2005; Niture et al., 2005; Chen et al., 2007) (**Figure 3**).

Among respiratory enzymes, in plants only GLDH is sensitive to oxidative stress induced by H_2_O_2_ (Leferink et al., 2009) due to the modification of Cys-340 presumably *via* reversible S-glutathionylation. In this way, glutathione (GSH) may protect the enzyme from overoxidation allowing production of ASC. However, in animal systems, the glutathionylation of mitochondrial respiratory proteins such as complex I, II, ATP synthase, and COX have been described as a modulator of mitochondrial redox status (Fratelli et al., 2003; Chen et al., 2007; Hurd et al., 2008; García et al., 2010) (**Figure 3**).

Related to glutathionylation of photorespiratory enzymes in mitochondria, the inhibition of GDC-P subunit activity by glutathionylation has been described by Palmieri et al. (2010) using partially puriﬁed P subunit from Arabidopsis leaf mitochondria. The fact that NO is also able to modulate GDC activity suggests the possibility of certain competitive effect of S-glutathionylation and S-nitrosation which may depend of the susceptibility of each protein to undergo these modifications (**Figure 3**).

Some possible targets of TRXs has been identified as glutathionylated in mammals, so the overlap between regulation by TRX and glutathionylation may be important to better understand the complex network of redox-regulated processes in plant cells (Gao et al., 2009). Human thioredoxin was identified as glutathionylated and the modification likely decreased its activity (Casagrande et al., 2002). In plants, Arabidopsis chloroplastic TRX*f* was identified as glutathionylated (Michelet et al., 2005) and interestingly, thiolation of poplar mitochondrial TRX*h*2 in an additional cysteine not belonging to the active site was shown to increase the redox potential of the enzyme (Gelhaye et al., 2004) (**Figure 3**). Also, PRXs undergo glutathionylation as in pea chloroplastic 2-Cys PRX in which the glutathionylation of the decameric form induced a change to its dimeric glutathionylated form (Calderón et al., 2017b) or in the poplar 1-Cys PRXIIB with a conformational dissociation of homodimers into monomers (Noguera-Mazon et al., 2006). This suggested the existence of a redox-dependent oligomerization switch in the PRX family by thiolation although it does not occur for all PRXs: this is the case of mitochondrial PRXIIF in which glutathionylation did not provoke a change in its oligomeric state, being GSSG able to glutathionylate both peroxidatic (Cys59) and resolving (Cys84) cysteine, provoking a decrease in the peroxidatic activity of the protein (Calderón et al., 2017b) (**Figure 3**). Interestingly, pea 2-Cys PRX and PRXIIF were differentially sensitive to small changes in GSH/GSSG ratio pointing to a fine regulation by the intensity of the oxidative stress. This could be a key step during cellular metabolism but mainly during stress conditions modulating the peroxidase activity and thus affecting H_2_O_2_ signaling. Both pea chloroplastic 2-Cys PRX and mitochondrial PRXIIF are regenerated from their overoxidized forms by pea SRX, which is then reduced by TRX (Iglesias-Baena et al., 2010; Iglesias-Baena et al., 2011). Interestingly, SRX deglutathionylated pea 2-Cys PRX but not PRXIIF, so the glutathionylation/deglutathionylation processes may have an important role during plant development and response to stress in which redox changes influence the posttranslational regulation of key proteins as the ones involved in the TRX/PRX/SRX system.

### Targets of S-Nitrosation

A large number of mass spectrometry (MS)-based analytical methods has been developed to face the challenge of analyzing S-nitrosylated proteins. However, the most often-used method is the biotin-switch assay (BST) (Jaffrey and Snyder, 2001). This methodology includes firstly the blocking of all the free thiols groups of cysteine residues, then selective reduction of modified cysteine and switch with a stable functional group usually with a biotin molecule that also serves as a handle for enrichment and detection. Despite this method is time consuming, tedious, and unsuitable for monitoring dynamic changes of this PTM *in vivo* (Feng et al., 2019), it has allowed to gain insight into the processes affected by S-nitrosation and into the study of the mechanism and effect that this PTM has on protein functions. Recently, newer strategies have been reported to overcome these issues. A S-nitrosoproteomic analysis using iodo-TMT (iodo-tandem mass tag) labelling, affinity enrichment, and high-resolution LC-MS/MS has been used to identify S-nitrosated proteins in tea plants (Qiu et al., 2019). Also, in animals, a new chemical proteomics strategy for quantitative analysis of reversibly modified cysteine using bioorthogonal cleavable-linker and switch technique (Cys-BOOST) has been reported. This method shows a higher sensitivity and considerably higher specificity and precision than other methods. It allows the proteome wide identification of SNO even from low abundance proteins and under basal conditions (Mnatsakanyan et al., 2019).

Similar to glutathionylation, several TCA enzymes including ACO, MDH, ICDH, α-ketoglutarate dehydrogenase, FUM, CS, and pyruvate dehydrogenase have been found to be S-nitrosylated in several plant species using different methodological approaches (Fares et al., 2011; Puyaubert et al., 2014; Hu et al., 2015; Lindermayr et al., 2015; Qiu et al., 2019) (**Figure 3**). However, the mechanisms by which this PTM happens, how S-nitrosation affects the enzymes activity or how S-nitrosation is affected by stress conditions are not well known. Previously it has been reported that ACO is reversibly inhibited by NO by a mechanism which promotes the loss of the iron-sulfur cluster, which can subsequently be reassembled under the proper conditions (Drapier, 1997). Recently it has been also shown that this inhibition under hypoxia results in accumulation of citrate, the latter in turn induces AOX and causes a shift of metabolism towards amino acid biosynthesis (Gupta et al., 2012). Regarding MDH, it has also been found to be S-nitrosated in peroxisomes which activity has been shown to be inhibited by NO donors (Ortega-Galisteo et al., 2012). Additionally, ICDH has been reported to be slightly over-nitrosylated in Arabidopsis cell suspension submitted to salt stress (Fares et al., 2011). In plants, the effect of S-nitrosation on ICDH has not been described unlike in animals, where this PTM leads to an inactivation of the enzyme and to a pro-oxidant condition in the cell (Yang et al., 2002; Lee et al., 2003).

SDH in plants is susceptible to S-nitrosation *in vivo* (Fares et al., 2011; Hu et al., 2015; Qiu et al., 2019) (**Figure 3**) under normal but not under saline stress conditions (Camejo et al., 2013). To our knowledge, in plants the effect of the S-nitrosylation in SDH activity is not known, although an inhibitory effect has been described in animals (Rizza et al., 2016).

Among the enzymes involved in the photorespiratory process, GDH has been reported to be S-nitrosated *in vivo* in Arabidopsis (Palmieri et al., 2010; Puyaubert et al., 2014; Hu et al., 2015) (**Figure 3**). As previously mentioned, *in vitro* studies have shown the effect of S-nitrosation on GDC-P subunit activity (Palmieri et al., 2010). Additionally, our laboratory has also found that in pea, both subunits P and T of GDC showed the same S-nitrosation pattern in control plants and salt-treated plants, with no changes in protein levels during plant development and salt stress, although loosing S-nitrosation in older plants (Camejo et al., 2013). S-nitrosation of SHMT has been reported by Camejo et al. (2013); Hu et al. (2015) and Tanou et al. (2009) in Arabidopsis, pea, and citrus plants, respectively (**Figure 3**). Despite of previous report where photorespiration has been shown to increase under salinity (Hoshida et al., 2000), our lab has reported that SHMT did not change its protein levels during salt stress although it was found S-nitrosated in control pea mitochondria but not in those from stressed plants. We hypothesized that its denitrosation after long period of salt stress may allow photorespiration to be functional under these conditions.

Among the RNS targets in plant mitochondria are some ETC components, with NO inhibiting cytochrome c pathway whereas AOX is only partially inhibited (Martí et al., 2012). The different inhibitory effect may be involved in the regulation of ROS generation and energy metabolism in the organelle collaborating in the stress response. In fact, the reported effect of NO on different enzymes components of the antioxidant system as the lack of inhibition on Mn-SOD or on the majority of the ASC-GSH cycle except APX in pea plants could be part of a NO redox signalling through the H_2_O_2_ and NO cross-talk (Martí et al., 2012). However, similar to AOX, COX has not been found to be S-nitrosated in plants according to the references checked for this review, although it has been reported to be S-nitrosated in animals (**Figure 3**) (Zhang et al., 2005).

Under both, normal and stress conditions, PRXs from animals and plants have been shown to be targets of S-nitrosylation (**Figure 3**). In Arabidopsis and citrus plants under normal and salinity conditions, respectively, PRX is one of the proteins reported to be S-nitrosated (Tanou et al., 2009; Lindermayr et al., 2015). Deeper studies have also shown that S-nitrosation of plants PRXs leads to a change in their protein activity. In this sense, in chloroplasts, both the hydroperoxide-reducing peroxidase activity during the plant hypersensitive disease resistance response and the ONOO− detoxification activity of PRXIIE are inhibited by S-nitrosation (Romero-Puertas et al., 2007; Romero-Puertas et al., 2008). Recently, mitochondrial PRXIIF has been found by our lab to be S-nitrosated *in vivo* under long saline stress conditions, a situation in which NO levels were also increased (Camejo et al., 2013). The effect of NO and S-nitrosation on the protein function has been further investigated in our lab (Camejo et al., 2015). Under *in vitro* conditions, we found that both catalytic cysteines of PsPRXIIF (C59 and/or C84) are susceptible of S-nitrosation depending of its oligomerization state. We also found that the S-nitrosation leads to a conformational change that inhibits the PsPRXII peroxidase activity in favour of the transnitrosylase activity. These data together with the previous finding of PRXIIF being nitrosated only under long saline conditions and the reversibility of S-nitrosation and, consequently, of its peroxidase activity, suggest that the S-nitrosation of PRXIIF might act as a mechanism that is activated under oxidative and nitrosative stress, a situation that is presented in long saline conditions and that is reversed by reducing conditions, in which the TRX system may function in the mitochondria.

The level of S-nitrosated proteins depends on NO levels in the cells, which are regulated by several mechanisms. They depend on GSNO which is regulated by GSNOR activity (Liu et al., 2001; Holzmeister et al., 2011) and on Tyr nitration of proteins which consumes NO. Another important mechanism affecting S-nitrosation state of proteins may be through TRX activity, which similarly to GSNOR, can degrade S-nitrosothiols to increase their turnover ratio (Benhar et al., 2008). Several S-nitrosated enzymes that are summarized in this review have been also shown to be targets of mitochondrial TRX (see review in Møller et al., 2020). From plant S-nitrosation studies under abiotic stresses (Fares et al., 2011; Camejo et al., 2013; Puyaubert et al., 2014), it could be suggested that under those conditions, the effect of the stress does not produce large changes on the S-nitrosation status of the cells, probably due to other mechanisms that could be competing with the S-nitrosation process. Therefore, in the future, it might be more important to answer which cysteine residues in a specific protein are differentially S-nitrosated under normal and stress conditions and in which proportion, and also, which are the biochemical mechanisms involved.

### Targets of Persulfidation

The chemical reactivity of H_2_S provokes the modification of cysteine residues to form persulfides, a PTM that may cause functional changes in protein structures and activities.This modification has been reported to increase under oxidative stress and it has been postulated as a defense mechanism against protein oxidative damage, avoiding the formation of dangerous -SO_2_H or irreversible -SO_3_H. Trying to identify the target proteins of persulfidation, a few works have been carried out in plant extracts. In a first approach, using a modiﬁed BST with S-methyl-methanothiosulfonate (MMTS) to block free thiols and the thiol-speciﬁc biotinylating agent biotin-HPDP, 106 proteins were identified as persulfidated in Arabidopsis mainly involved in photosynthesis, protein synthesis, and cell organization (Aroca et al., 2015). More recently, using methylsulfonylbenzothiazole (MSBT) to block both thiols and persulﬁde groups and cyanoacetate-based reagent CN-biotin as labelling agent, more than 3,000 proteins were identified as possible targets in cytosol-enriched leaf extracts of Arabidopsis plants grown under physiological conditions (Aroca et al., 2017). The bioinformatic analysis revealed that persulfidated cysteines are involved regulating important processes such as plant growth, plant response to abiotic and biotic stresses, carbon metabolism, and RNA translation. Several mitochondrial proteins were described as possible targets of persulfidation, including among others, proteins involved in ATP synthesis, respiration, chaperone function, protein synthesis, and all the TCA and photorespiratory enzymes (**Figure 3**). Interestingly, TRX*o*1 and almost all the described PsTRX*o*1 targets (Martí et al., 2009) were also found as persulfidated (ATP synthase subunit alpha, thiosulfate/3-mercaptopyruvate sulfurtransferase, elongation factor Tu and PRXIIF), implying that the role of this mitochondrial TRX*o*1 is related to persulfidation as it has been described for other TRXs (Filipovic and Jovanović, 2017) (**Figure 3**). Some of them were also demonstrated as S-nitrosylated and/or glutathionylated as described above, that reinforces the role of these PTMs in the processes in which the proteins are involved in mitochondria.

Another mechanism of action of H_2_S independent of its persulfidation effect is its coordination with the metal center of metalloproteins, attaching covalently to heme porphyrins. In this way, H_2_S acts as a potent inhibitor of plant and animal COX. Additionally, both COX and NADH dehydrogenase have been found among the persulfidated proteins in Arabidopsis (Aroca et al., 2017) (**Figure 3**).

A beneficial effect of exogenous application of H_2_S on plants subjected to different abiotic stresses has been described (see review by Corpas and Palma, 2020), including heavy metals, arsenic, low and high temperature, salinity, or drought. In fact, a reduction of oxidative stress on lipids and proteins as well as an increase in antioxidant components have been observed in the treated plants, contributing to a better response under the stress situation. However, to our knowledge, the effect on specific mitochondrial targets of persulfidation is scarcely reported.

### Cross Talk Among H_2_S and Reactive Oxygen and Nitrogen Species

The crosstalk between H_2_S and RNS/ROS is important for the biological functions of all these molecules (Scuffi et al., 2014; Corpas et al., 2019b). H_2_S can act as a reductant reacting with biological oxidants, such as NO^·^, H_2_O_2_, O_2_^·−^, peroxynitrite, hypochlorite, and S-nitrosothiols although the direct reactions have not been quantified in plants (Gotor et al., 2019). Several examples point to the existence of the mentioned crosstalk. It has been described that H_2_S enhanced antioxidant capacity and salt tolerance of cucumber hypocotyls and radicles (Yu et al., 2013). The overlap between persulfidation and sulfenylation has been described by proteomic studies [reviewed by Zhou (2020)], with 437 proteins being modified by both. However, the effect of each modification can be opposite. As examples, activities of glyceraldehyde- 3-phosphate dehydrogenase (GAPC) and cytosolic APX are inhibited by sulfenylation and increased by persulfidation (Kitajima et al., 2008; Zaffagnini et al., 2013; Aroca et al., 2015), pointing to a fine regulation of the proteins by different PTMs. Related to RNS, an increase in NO occurs after H_2_S treatments of nitrate-treated tomato (Guo et al., 2018). Also, among 927 S-nitrosated proteins in Arabidopsis, 639 may be persulfidated (Hu et al., 2015; Aroca et al., 2018) with different functions on specific targets: both PTMs increased cAPX activity while S-nitrosation inhibited GAPC and persulfidation increased it (Begara-Morales et al., 2014; Lindermayr et al., 2015; Yang et al., 2015). As another example, exogenous treatments of H_2_S and NO alleviated some abiotic stresses and maintained the quality of post-harvested fruits (Ziogas et al., 2015; Corpas and Palma, 2018). Although the cascade of action is not fully elucidated, some experiments have reported that H_2_S could act upstream of NO signaling during stomatal closure while downstream in response to abiotic stress (Scuffi et al., 2014).

All these examples evidence that the interaction among H_2_S, RNS, and ROS may occur to regulate physiological processes not only under control conditions during plant development but also under stressed environments, allowing signal transduction pathways through the posttranslational regulation of key proteins involved in the response. The identification of the target proteins of each modification driven by H_2_S, RNS, and ROS and their effects on enzyme structure and activity are essential to understand the complexity of interactions and will help to reveal the modulating effect of redox components.

## The TRX-PRX System Under Stress Conditions

Stress conditions provoke changes in cellular redox homeostasis mainly by dangerous increase in ROS/RNS generation (Noctor et al., 2015; Calderón et al., 2018a). APX, a component of the ASC-GSH cycle together with monodehydroascorbate reductase (MDHAR), dehydroascorbate (DHA) reductase (DHAR) and glutathione reductase (GR), is the responsible of H_2_O_2_ scavenging in mitochondria (Jiménez et al., 1997; Locato et al., 2018). Also, GPX, GRX, and TRX/PRX system play an important role in cellular ROS homeostasis. In this context, either mitochondria as chloroplasts are essential in maintaining the cellular redox balance under stress conditions (Cejudo et al., 2014; Sevilla et al., 2015b; Ojeda et al., 2017).

**Figure 4:**
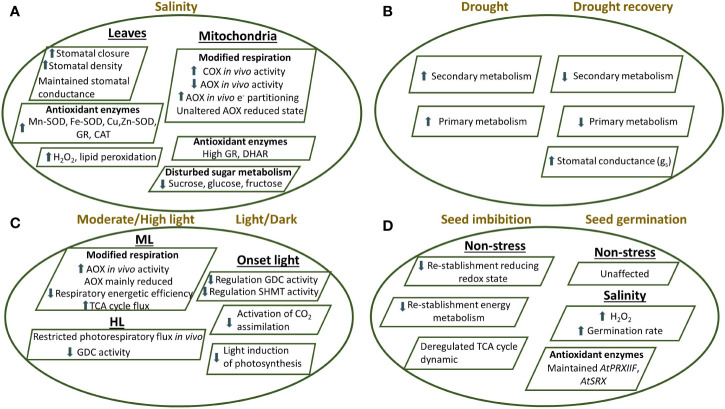
Proposed model showing the response of *Arabidopsis thaliana* plants knockout of the mitochondrial TRX*o*1 under different stress conditions as **(A)** salinity in leaves and mitochondria, **(B)** drought and drought recovery, **(C)** moderate light/high light and **(D)** during seed imbibition and germination, as detailed in the main text (Daloso et al., 2015; Ortiz-Espín et al., 2017; Calderón et al., 2018b; Florez-Sarasa et al., 2019; Sánchez-Guerrero et al., 2019; Da Fonseca-Pereira et al., 2019b; Da Fonseca-Pereira et al., 2019c). Effect on stomatal behaviour, antioxidant and oxidative metabolism, respiration and photorespiration, photosynthesis, TCA cycle as well as primary and secondary metabolism are shown in the KO plants compared to non-transformed plants. AOX, alternative oxidase; CAT, catalase; COX, cytochrome oxidase; Cu/Zn-SOD, copper-zinc superoxide dismutase; DHAR, dehydroascorbate reductase; GDC, glycine decarboxylase; Fe-SOD, iron superoxide dismutase; GR, glutathione reductase; Mn-SOD, manganese superoxide dismutase; PRXIIF, peroxiredoxin IIF; SHMT, serine hydroxymethyl transferase; SRX, sulfiredoxin; TCA, tricarboxylic acid.

Changes in redox state and its significance in cellular signaling process can be evaluated in plant tissues using different approaches. Mutants are useful tools for investigating the involvement of specific proteins *in vivo* on redox metabolism in plant cells. Also, a parallel approach is to analyze stress conditions affecting the intracellular redox balance. In this sense, loss of function *trxo*1*-1* and *trxo*1*-2* mutants have been used not only to corroborate a role for the TRX system in regulating different metabolic processes in mitochondria but also to define the importance of mitochondrial TRXs under stress conditions (Daloso et al., 2015; Calderón et al., 2018a). However, in these studies, no extreme phenotype has been described, either in standard or stress conditions, possibly due to the redundancy or overlapping functions with mitochondrial or cytosolic proteins as other TRXs and GRXs (Daloso et al., 2015; Geigenberger et al., 2017; Ortiz-Espín et al., 2017; Calderón et al., 2018a; Calderón et al., 2018b; Sánchez-Guerrero et al., 2019). The information related to the physiological mitochondrial TRX*o*1/TRX*h*2 function in plant stress acclimation is scarce. *In silico* studies on *AtTRXo1* gene expression have revealed that it does not vary as a response to different environment conditions including salinity. Contrary, in pea plants, an adaptative response to a short-term high salinity levels (150 mM NaCl), including increased *PsTRXo1* gene expression was demonstrated, while longer salinity treatment downregulated it, with a parallel increase in PsTRX*o*1 protein and activity. Interestingly, these TRX*o*1 related changes were correlated with an increase in the AOX capacity in purified mitochondria preparations as well as a higher demand to regenerate the oxidized form of PRXIIF involved in ROS detoxification. Also, a maintained *MnSOD* gene expression, protein and activity levels were observed. Overall, the results demonstrated the participation of PsTRX*o*1 as a component in the mitochondrial antioxidant response allowing plant salinity adaptation (Martí et al., 2011). In order to gain further insight into the physiological and metabolic function of the mitochondrial TRX*o*1 in the plant acclimation to saline stress, we have recently used two independent mitochondrial *Attrxo1* mutants (*Attrxo1-1, Attrxo1-2*) (Calderón et al., 2018b). Their responses to salinity fit well with those reported by Martí et al. (2011) in pea plants, all pointing that TRX*o*1 is required for the proper functioning of the antioxidant metabolism including compensatory mechanisms, as higher levels of H_2_O_2_ and NO, an upregulation of all SOD isoforms, catalase and GR, alterations in the glutathione redox state, due to maintained GSH content in *Attrxo1* plants and changes in stomatal density, stomatal closure with lower water loss, which may be also a key factor for the adaptative response to salinity (Calderón et al., 2018b) (**Figure 4**). At mitochondrial level, a more recent study has revealed the impact of the lack of TRX*o*1 in the acclimation to salinity, with an important *in vivo* reorganization of the respiratory and antioxidant enzymes as well as metabolic responses (Sánchez-Guerrero et al., 2019). Compelling evidences from several plants indicated that *AOX* transcript and protein increase during salinity (Smith et al., 2009; Martí et al., 2011; Lázaro et al., 2013; Del-Saz et al., 2016). Using *Attrxo1-1* and *Attrxo1-2* mutants under a long-term saline stress, we described that AOX protein displayed a reduction of its *in vivo* activity in all genotypes and that exits a higher electron partitioning to the AOX pathway under salinity which denotes a relatively higher response of the AOX that can act preventing the generation of O_2_^.-^at the UQ level (Purvis, 1997; Cvetkovska and Vanlerberghe, 2012). In parallel, the high constitutive mitochondrial GR and DHAR activities in At*ttrxo1* mutants, could act helping to decrease H_2_O_2_ content and to increase GSH recycling. Importantly, we observed a change in AOX isoforms pattern but AOX protein was not decreased and was also maintained in its reduced state under control and saline conditions in both *Attrxo1* mutants. These observations suggested that *in vivo*, the mitochondrial TRX*o*1 system could perform a maintenance of reductive function rather than an AOX regulation, as it has been proposed. This possibility was also suggested recently by Nietzel et al. (2017) (**Figure 4**). Furthermore, a pronounced decrease on glucose and fructose levels occurred in both *Attrxo1 mutants* under control and salinity, coinciding with an increased *in vivo* respiration through the cytochrome c (COX) pathway (Sánchez-Guerrero et al., 2019). These results indicated a reorganization in central carbon metabolism and reflect a higher use of these sugars in the glycolytic pathway, causing an increased respiration, probably driven by an increased carbon flow through the TCA cycle as previously suggested (Daloso et al., 2015). The increase in ATP-coupled respiration is an indicator of a higher demand on the leaf energy of the mutants under control conditions (**Figure 3**).

A similar metabolic adjustment could be also taking place during seed germination, a process in which a function for AtTRX*o*1 has also been described (Ortiz-Espín et al., 2017). Arabidopsis *AtTrxo1* transcript levels were particularly high in dry seeds and cotyledons, where it reached a maximum coinciding with 50% germination. However, expression was lower in seeds germinating under salinity. We reported that seeds of both *Attrxo1-1* and *Attrxo1-2* mutant lines failed to show any important difference in the germination rate compared to WT, but the physiological and metabolic analysis of these mutants showed diverse and complex responses, showing higher H_2_O_2_ levels in dry seeds. Moreover, A*ttrxo1* mutant seeds germinated faster and accumulated higher H_2_O_2_ content under salinity (**Figure 4**). This peak of H_2_O_2_ at the beginning of germination might be a factor in the observed early germination rate and fits well with that proposed for the accumulated mitochondrial H_2_O_2_ at early stages of germination (Zhang et al., 2014). Thus, a role for AtTRX*o*1 in redox homeostasis during seed germination, acting as a possible sensor of saline stress and/or an inducer of H_2_O_2_ accumulation was proposed. This specific role of TRX*o*1 under stress may in turn be related to its targets following seed germination, an aspect that deserves further investigations (Ortiz-Espín et al., 2017).

Recently, the earliest events in energy and redox metabolism of Arabidopsis seeds at imbibition, as a physiological step of metabolic reactivation, have been investigated (Nietzel et al., 2020). RoGFP-based *in vivo* sensing experiments, have suggested that the reestablishment to a more reducing thiol redox status of the mitochondrial matrix and the cytosol within minutes in intact seeds, is intimately linked to the reestablishment of energy metabolism. Redox proteomic analysis has shown that active site cysteine peptides of GR 2, NTR a/b, and TRX*o*1 present the strongest change in redox state. Seeds germination of the three mutants *gr2, ntra/b*, and *trx-o1* was associated with increased respiratory rates and deregulated TCA cycle dynamics, suggesting decreased resource efficiency of energy metabolism. NAD-malic enzyme (NAD-ME) and aconitase (ACO) also showed a quantitative redox-dependent response, but this was not the case for enzymes as 2-oxoglutarate dehydrogenase (OGDH), NAD-isocitrate dehydrogenase (ICDH), and ICDH which only showed a marginal response at imbibition. These differences indicated that the functional impact of cysteine redox switch operation is enzyme-specific. An important contribution of redox regulation to efficient metabolism during early seed germination was proposed.

The importance of the mitochondrial NTR/TRX system under drought episodes was recently investigated using both *Attrxo1* mutant and At*ntra ntrb* double mutant plants (Da Fonseca-Pereira et al., 2019b). Under these conditions, all the genotypes lacking functional NTR/TRX system, showed enhanced drought tolerance. Interestingly, *TRXo1* transcript was more highly expressed under drought, an effect even stronger mainly during repetitive drought/recovery events. Results on the changes in secondary metabolites with a large number of metabolites increasing in at least one of the *Attrxo1* mutants following two cycles of drought, reinforces the idea that secondary metabolism is redox regulated by TRX system (**Figure 4**). Finally, the analysis of the increased stomatal conductance following drought recovery suggested a TRX*o*1 redox regulation of stoma function (**Figure 4**) which fits well with the previously reported involvement of TRX*o*1 mediated redox regulation of stomatal dynamic and function under salinity (Calderón et al., 2018b).

Regarding the effects of different light conditions, it has been observed that an altered *in vivo* AOX activity and carbon metabolism occur in both Arabidopsis *trxo1* mutants under medium light (ML) and high light (HL) conditions (Florez-Sarasa et al., 2019). Contrary to the effects under salinity (Sánchez-Guerrero et al., 2019), the results showed that the *in vivo* AOX activity was higher in the *Attrxo1* mutants at ML while the AOX redox state was apparently unaltered as we have commented above. Moreover, the authors claim that the negative regulation of the TCA cycle by the TRX system is coordinated with the increased input of electrons into the AOX pathway. Under HL conditions, AOX and photosynthesis displayed similar patterns in the mutants. Furthermore, changes on photorespiration were observed under HL conditions, being restricted at the level of glycine decarboxylation, most likely as a consequence of the redox imbalance. These results denote the relevance of TRX*o*1 on the interaction between mitochondrial redox and carbon metabolism under light stress conditions (**Figure 4**). In a parallel study, the alteration of photorespiratory activity in the absence of TRX*o*1 was described. In this sense, it has been shown that a functional TRX*o*1 allowing the rapid induction of mitochondrial steps of the photorespiration process *via* GDC system in conjunction with SHMT is necessary to facilitate light-induction of photosynthesis (Reinholdt et al., 2019b).

**Figure 5:**
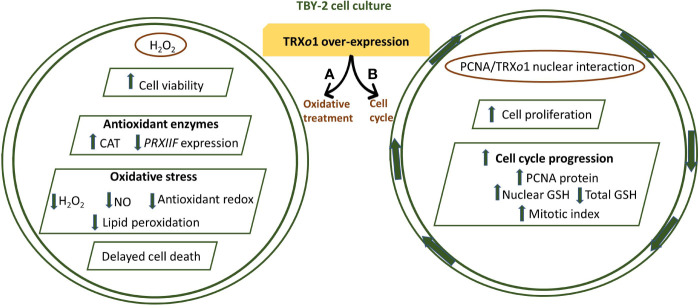
**(A)** PsTRX*o*1 function in cell response to high H_2_O_2_ treatment. TBY-2 cell lines over-expressing PsTRX*o*1 showed higher and maintained viability than control lines. The high catalase (CAT) activity, decrease in ASC and GSH redox state are parallel and might contribute to decreased H_2_O_2_, NO, and lipid peroxidation, protecting together with TRX*o*1, against increased oxidative stress, thus allowing delayed programmed cell death (PCD) (Ortiz-Espín et al., 2015). **(B)** Proliferating cell nuclear antigen (PCNA) interacts with PsTRX*o*1 in the nucleus and showed an efficient *in vitro* disulfide reductase activity reducing oxidized PCNA. Flow cytometry showed that TRX*o*1 increased the rate of cell proliferation and cell cycle progression. Higher amount of PCNA protein in over-expressing TRX*o*1 cells occurred in the growth phases when high mitotic index and percentage of cells in S-G2/M phases were more evident and different to that in control lines. These changes were parallel to maintained GSH level in the nucleus but decreased in total cellular GSH in over-expressing lines, when the percentage of cells in G2/M phase was higher. Overall, these changes suggest that TRX*o*1 is involved in cell cycle progression providing a reductive environment and interacting with PCNA (Calderón et al., 2017a).

Some other TRX*o*1 functional studies were focused on determining the effect of high *PsTRXo1* expression on processes linked to oxidative treatment and on the functional PsTRX*o*1 role in the nucleus. Under a situation of high H_2_O_2_ level treatment, the analysis of different hallmarks of programmed cell death (PCD) demonstrated that the over-expression of PsTrx*o*1 in tobacco (*Nicotiana tabacum*) BY-2 cells, was able to increase the cell viability in that oxidative situation, contrasting with a severe decrease in viability and marked oxidative stress, with a rapid cell death, observed in non-overexpressing lines. The decreased content in endogenous H_2_O_2,_ an increased catalase activity, with the practically maintained *PRXII* expression, would be involved in the delayed cell death found in over-expressing cells, in which changes in oxidative parameters and GSH/ASC redox state were less extended after the H_2_O_2_ treatment, than in non-overexpressing lines. These data pointed to PsTRX*o*1 as an important factor responsible for the delay in the PCD provoked by the oxidative treatment (Ortiz-Espín et al., 2015) (**Figure 5**). In pea leaves TRX*o*1 is constitutively found in mitochondria and nucleus (Martí et al., 2009). Nuclear localization has been reported for several cytosolic TRX*h* isoforms but only during oxidative stress (Pulido et al., 2009). While subsequent reports describing a functional TRX system in the plant nucleus have appeared, little evidence on the nuclear TRXs targets *in vivo* has been reported and only a few candidate protein targets have been experimentally validated (Delorme-Hinoux et al., 2016). Among them, proliferating cellular nuclear antigen (PCNA) has been demonstrated to interact with PsTRX*o*1 in the nucleus (Calderón et al., 2017a). In *Medicago* and barley seed embryos, PCNA was also reported as a candidate targets of TRX*h*, but the interaction was not conclusively demonstrated (Alkhalﬁoui et al., 2007; Hägglund et al., 2008). In TBY-2 cells it was reported that the over-expressed PsTRX*o*1 was localized in mitochondria and nucleus and the over-expression correlated with changes in the growth of the culture, increase in the rate of cell proliferation and a decrease in the total cellular GSH content, but maintained nuclear GSH accumulation (Calderón et al., 2017a). Changes in GSH content were accompanied by a higher mitotic index, unlike non-expressing TBY-2 cells. All these findings suggest that TRX*o*1 is involved in the cell cycle progression of TBY-2 cultures, possibly through its link with both GSH and PCNA (**Figure 5**).

The information on the involvement of the mitochondrial PRXIIF in stress is scarce, being the chloroplastic and cytosolic isoforms more studied. Among ROS sensors during stress, reactive thiols may play a key role in the signal transduction, and as example, the action of SRX on the oxidized PRX has been reported (Lázaro et al., 2013). A link between redox changes and gene regulation has been described during the stress response (Astier et al., 2011) being *AtPRXIIF* one of the responsive genes in oxidative-induced stress situations such as treatments of Arabidopsis cell cultures with H_2_O_2_, menadione or antimycin A (Sweetlove et al., 2002) or in pea and poplar leaves under cold stress (Barranco-Medina et al., 2007; Gama et al., 2007). Also under salinity and Cd stress in pea leaves but not in roots, both transcripts and protein levels were upregulated (Barranco-Medina et al., 2007), while in more resistant species as Arabidopsis, the leaf mRNA level did not change significantly under salt, ozone, H_2_O_2_, or light stress (Horling et al., 2002; Horling et al., 2003). In another study, PRXIIF presented a biphasic response in pea plants under salinity, being the gene upregulated after 5 days of 150 mM NaCl treatment while downregulated after 14 days (Martí et al., 2011). However, the protein content remained constant although a regulation by S-nitrosylation was described affecting structure and function as commented above (Camejo et al., 2013; Camejo et al., 2015). Also, under these conditions salinity provoked a change in the oligomerization pattern of PRXIIF, increasing the oligomeric forms of the protein, similar to that occurring when recombinant protein was treated with H_2_O_2_ (Camejo et al., 2015). Also, the analysis of mutants lacking AtPRXIIF has allowed the knowledge of the physiological role of the protein not only under control (where a compensatory mechanism by mitochondrial antioxidant system seems to occur) but also under stress situations (Lázaro et al., 2013). The mutants’ behaviour has revealed a role for PRXIIF in root growth under Cd stress in Arabidopsis seedlings (Finkemeier et al., 2005), water stress in *Vitis vinifera* and in Arabidopsis mature leaves (Gama et al., 2007; Vidigal et al., 2013). All these examples imply the important involvement of PRXIIF in the antioxidant defence and redox signalling in plant cells.

## Conclusion and Perspectives

In the last years, the advance in the analysis and characterization of TRX*o*1 mutants has allowed to obtain wide information on the function of this protein, unveiling the key processes by which TRX system regulates mitochondrial respiration, TCA cycle, photorespiration, plant acclimation to stress through a proper functioning of the antioxidant metabolism, as well as its link with the germination process and cell cycle progression. In general, in the scarce studies describing processes as germination and plants acclimation under different conditions, the lack of TRX*o*1 generates a decrease in the energetic efficiency, but the connection between TRX*o*1 and *in vivo* respiratory AOX, needs to be explored in more detail. Also, the compelling evidences that both, TRX*h*2 and *o*1 systems play a role in the redox regulation of the mitochondrial photorespiratory metabolism needs to be further investigated. Another interesting point is the possible existence of compensatory and redundant mechanisms as the existence of other TRX isoforms. The use of multiple mutants in different redox systems present in the cellular compartments will aid to unravel their functionality in which, the PTMs may play a key role. Understanding which is the signature of the signal that triggers the specific PTM associated to a specific stress and also how plants distinguish these signals, will help to get insight on how plants responds to the different environmental cues. Hopefully, the development of new mass-spectrometry proteomic techniques will allow in the near future to expand the knowledge on the dynamic and interaction among the different PTMs. This is important not only for fundamental research but also for crop improvement in the actual climate-changing environment.

## Author Contributions

MM, AJ, and FS conceived the idea and wrote the manuscript.

## Funding

The review was funded by a Saavedra Fajardo 20402/SF/17 (Fundación Séneca, Murcia, Spain) and a Ramón y Cajal (Ministry of Science and Innovation, Spain) Fellowship both awarded to MM and Spanish grants MINECO/FEDER (BFU2017-86585-P) and Seneca Excellence Project (19876/GERM/15).

## Conflict of Interest

The authors declare that this review article was written in the absence of any commercial or financial relationships that could be construed as a potential conflict of interest.
